# 

**Published:** 1892-07-23

**Authors:** 


**Notices of Births, Deaths, and Marriages,** are inserted in this column at a charge of 2s. 6d. for an announcement not exceeding four lines.

### NOTICE TO CORRESPONDENTS.

All MS., Letters, Books for Review, and other matters intended for the Editor should be addressed The Editor, The Lodge, Porchester Square, London, W.

The Editor cannot undertake to return rejected M.S., even when accompanied by stamped directed envelope.

**Notice to Advertisers and Subscribers.**

All Advertisements, Orders for Copies of The Hospital, and Business Communications should be addressed to The Publisher, *not to the Editor*, The Hospital, 140, Strand, W.C.
The Cost of Subscription, post free, is as follows:—Per week, 2½d.; per quarter, 2s. 6d.; per half-year, 5s.; per annum, 8s. 6d. Foreign:—Per annum, 10s. 8d.; payable, in all cases, in advance.

**"Burdett's Hospital Annual," 1891—1892.**

Third year of publication. 600 pp. Boards, gilt lettering. A most complete guide to British and Colonial Hospitals, Dispensaries, Nursing and Convalescent Institutions, and Asylums. Price 3s. 6d. Published by The Scientific Press (Limited), 140, Strand, W.C.

**NOTICE.—The Index for Vol. XI. (ending March, 1892) is on sale at the Office of this Journal, price 2½d., post free.**

### SCRAPS AND GLEANINGS.

DIPHTHERIA has broken out at Heywood. Several deaths are reported.

THE Coventry Hospital Saturday Fund collection amounted this year to £91 16s. 10d.

HER MAJESTY THE QUEEN has sent £50 to the Lord Mayor of London for the St. John's Fire Relief Fund.

THE Grocers' Company have sent a cheque for £100 to the Hon. Alfred Lyttleton, for the Children's Country Holidays Fund.

BARON HIRSCH has forwarded a donation of £500 to the Committee of the Royal Hospital for Women and Children, in aid of their funds.

MR. WALTER I. CARNT, clerk to the Visiting Justices of H.M. Prison, Derby, has been appointed Secretary to the Derbyshire Royal Infirmary.

THE Queen has given directions for the appointment of Mr. Francis Voley Pougnet, M.D., to be a member of the Council of Government of the colony of Mauritius.

AT the Court of Common Council the Corporation voted £210 to the Mansion House Fund for the relief of the sufferers by the recent fire at St. John's, Newfoundland.

D. STUHLMANN, the companion of Emin Pacha on the latter's latest expedition, has arrived from the interior at the coast town of Bagamayo, in German East Africa.

THE new list of Doctors of Science of the University of London includes the name of Mr. Bernard Dyer, on whom the degree has been conferred for contribution to the Chemistry of Agriculture.

PRINCESS LOUISE, accompanied by the Marquis of Lorne, went by the steamer Palm last week to Greenwich, where she distributed the prizes gained by the boys of the Greenwich Hospital School.

THE Duchess of Albany has consented to present the certificates awarded to ladies, in connection with lectures on Domestic Hygiene, at a meeting of the Sanitary Institute to be held on July 27th.

NEWS has reached England of the sudden death of Dr. Gavin Russell, medical missionary, who four years ago went out to labour in Formosa in connection with the English Presbyterian Missionary Society.

M. PASTEUR is lying ill at his residence at Garches, near St. Cloud, from what is believed to be a mild attack of cholera, and, according to Reuter's correspondent, his condition gives rise to anxiety among his friends.

DR. CARLYLE, Langholm, Newcastle, has been publicly presented with his portrait (painted by Sir George Reid, President of the Royal Scottish Academy), the occasion being the completion of fifty years' medical practice in the town.

THE University College Hospital, Gower Street, and the North-West London Hospital, Kentish Town Road, have each been presented with a cheque for 25 guineas, collected by means of a church parade arranged by friendly societies in North London in May last.

A LANDSLIP occurred last week in the mountains above the baths of S. Gervais, the well-known mineral springs and summer resort in the gorge of the Bonnant, near Chamounix. Three houses were totally and one partially destroyed. Several people were killed.

A BOY named Butterfield, belonging to an excursion from Grimsby, strolled with a companion along the South Cliffs at Scarborough into a hayfield. A man called to them that they were trespassing, and in his haste to escape, Butterfield stumbled over the cliff and was killed.

THE medical staff and lecturers of the Dental Hospital recently gave a conversazione at the Prince's Hall, Piccadilly. A good musical entertainment was provided, and the Society of Portrait Painters' Exhibition was viewed by the guests in the Royal Institute Galleries of the hall.

THE *British Medical Journal* understands that Sir Trevor Lawrence will be elected treasurer of St. Bartholomew's Hospital on the resignation of Sir Sydney Waterlow, which will be formally accepted on July 28th. The new treasurer will not, it is believed, reside within the hospital.

THE Royston Cottage Hospital continues in good working order. The receipts for 1891, after deducting the balance brought forward and the loan, amount to £225 12s. 6d., while, on the other side of the account, the total expenditure is £243 7s. 1d., or an excess of £17 14s. 7d. over the receipts.

THE Duchess of Albany recently presented seventy-seven certificates to the successful candidates of the St. John's Ambulance Association at Streatham. She also witnessed a demonstration of the work under the direction of D Matthew Coates. A guard of honour was furnished by the 2nd V.B. East Surrey Regiment.

THE annual report of the Sarah Nicol Memorial Cottage Hospital and Male Convalescent Home states that the sum of £250 free of legacy duty had been received from Miss Hannah Pickard. Several other donations were also acknowledged. The hospital is doing a good work, and 112 cases were treated during the past year.

THE foundation-stone of the new Eye Infirmary for Sunderland and North Durham was laid by Mrs. Backhouse. The building is two storeys high, with four wards—two male and two female—providing accommodation for 16 in-patients. There is also accommodation for nurses and servants, besides two bath rooms and the usual offices.

THE new Cottage Hospital at Horsham was opened the beginning of the month, by the President, Mr. C. T. Lucas. The building is executed in red brick up to the first floor, and above this is half-timber work filled in with rough cast plaster, the front portions being hung with bright Sussex tiling. It has apparently been the architect's intention to produce a building of cottage-like appearance, but every modern requirement of a hospital has been provided.

IN 1883 Mr. W. Bradfield, of Islington, made his will, in which he bequeathed £1,000 to the British Hospital for Diseases of the Skin, Finsbury Square. When he died, in 1890, the institution in Finsbury Square, which was one of several branches, had been discontinued for six years. The residuary legatee claimed that the £1,000 fell into the residue. The dispute was carried before Mr. Justice Chitty, who held that the hospital was entitled to the legacy.

### THE STUDENT OF MEDICINE.

[All communications for this Supplement should be addressed to THE EDITOR of THE HOSPITAL, at 140, Strand, with the words, "Students' Column," written in the left hand top corner of the envelope.]

### APPOINTMENTS.

Alcock, Reginald, appointed Assistant House Surgeon to the North Staffordshire Infirmary and Eye Infirmary, Stoke-on-Trent. Collinson, Henry A., appointed House Surgeon to the Royal Infirmary, Newcastle-on-Tyne. Craig, Wm. Wallace, L.R.C.P.Lond., M.R.C.S.Eng., appointed House Surgeon to the Salop Infirmary, Shrewsbury. Crombie, J. Frank, M.B., C.M.Edin., appointed House Surgeon to the Great Yarmouth Hospital. De Renzi, Henry Castriot, M.R.C.S.Eng., L.R.C.P. Lond., appointed House Surgeon to the Westminster Hospital. Fagan, Joseph P., L.R.C.S.I., L.R.C.P. and L.M., appointed Assistant Surgeon to the South Dispensary, Liverpool. Farquharson, William F., M.B.Edin., Medical Assistant Borough Asylum, Exeter, late House Surgeon Dundee Infirmary, formerly Junior Medical Assistant Counties Asylum, Carlisle, appointed Assistant Medical Superintendent Counties Asylum, Carlisle. Folker, Henry Herbert, M.R.C.S.Eng., L.R.C.P.Lond., appointed Honorary Assistant Ophthalmic Surgeon to the North Staffordshire Infirmary and Eye Hospital, Stoke-on-Trent. Haworth, Stephen R., M.D., appointed House Surgeon to the Bridgnorth Infirmary. Holford, Walter S., L.R.C.P., M.R.C.S., L.D.S.Eng., appointed Dental Surgeon to the Belgrave Hospital for Children. Houghton, Murtaugh J., L.S.A., appointed Assistant to the House Surgeon and Physician Wolverhampton General Hospital. Mackintosh, Donald Jas., M.B., C.M.Glas., appointed Medical Superintendent of the Western Infirmary, Glasgow. Murray, R. W., F.R.C.S.Eng., appointed Surgeon to the Liverpool Infirmary for Children. Pearson, Joseph, M.B., C.M.Aberd., appointed Honorary Assistant Medical Officer to the Children's Hospital, Sheffield. Ross, Donald M. M., M.B., C.M.Ed., Senior Assistant Medical Officer Cumberland and Westmorland Asylum, Carlisle, appointed District Medical Officer in the Government Medical Service, Jamaica. Wait, John Alfred, B.A., M.B., B.C.Camb., appointed House Surgeon to the Clayton Hospital, Wakefield.

### VACANCIES.

The following vacancies are announced this week, the amount of salary in each case being given after the name of the institution:

**Resident Medical Officers**

Bury Dispensary Hospital, Bury, Lancashire, Senior House-Surgeon, doubly qualified—Salary, £100 per annum, with board, &c.
Fulham Union, Assistant Medical Superintendent of the Union Infirmary; doubly qualified; unmarried—Salary, £100 per annum, increasing £10 yearly to £130, with board, &c.
Great Northern Central Hospital, Holloway Road, N., House Physician—Salary, £60 per annum, with board, &c.
Great Northern Hospital, Holloway, N., House-Surgeon—Salary, £60 per annum, with board, &c.
Nottingham Borough Asylum, Mapperley Hill, Nottingham, Resident Clinical Assistant; appointment for six months—Board, &c.
Royal Portsmouth, Portsea, and Gosport Hospital, Assistant House-Surgeon, appointment for six months—Board, &c., and honorarium of £15 15s. at the expiration of term of office.
Royal Sea-bathing Infirmary for Scrofula, Margate, Resident Surgeon doubly qualified—Salary, £100 per annum, with board, &c.
St. Luke's Hospital, Resident Clinical Assistant, appointment for six months—Board, &c.
Sheffield General Infirmary, Assistant House Surgeon, doubly qualified—Salary, £80 per annum, with board, &c.
Victoria Infirmary of Glasgow, 22, Carlton Place, Glasgow, Superintendent and Resident Medical Officer—Salary £150 per annum, with board, &c.

### PASS LISTS.

**Royal University of Ireland.**

FACULTY OF MEDICINE.—The examiners have recommended that the undermentioned candidates shall be adjudged to have passed the First Examination in Medicine: Dora E. Allman, Queen's College, Cork; Emma F. A. Bailey, Royal College of Science, Dublin; S. T. Beggs, Queen's College, Belfast; H. J. Bell, B.A., Trinity College, Dublin; E. C. B. Carbery, Queen's College, Galway; S. Connor, Queen's College, Cork; P. Convery, University College, Dublin; J. Crean, University College, Dublin; H. Currell, Queen's College, Belfast; T. F. Dillon, Queen's College, Cork; R. V. Donelan, Queen's College, Galway; R. P. Farnan, University College, Dublin; G. K. Finlay, University College, Dublin; T. Finucane, Queen's College, Cork; S. F. Floyd, Queen's College, Belfast; H. J. Fryer, Queen's College, Belfast; F. Fulton, Queen's College, Belfast; T. K. Greenfield, Queen's College, Belfast; P. J. Hamilton, University College, Dublin; J. Harvey, Queen's College, Belfast; J. C. B. Hayes, Queen's College, Cork; J. Johnston, Queen's

THE STUDENT OF MEDICINE—(*continued*).

College, Belfast; W. G. Jordan, Queen's College, Belfast; J. Lennon, Queen's College, Belfast; J. F. Little, Queen's College, Belfast; A. A. F. McArdle, Queen's College, Cork; J. McArdle, University College, Dublin; W. A. McBroom, Que n's College, Belfast; C. C. McCarthy, University College, Dublin; A. L. McCully, Queen's College, Belfast; J. McElroy, Queen's College, Belfast; R. R. McLean, Queen's College, Belfast; J. J. A. G. McMurtry, Queen's College, Belfast; H. J. McNabb, University College, Dublin; L. T. Moore, Queen's College, Cork; W. W. Moore, B.A., Queen's College, Belfast; M. R. Morrissey, University College, Dublin; R. Morrow, Queen's College, Belfast; J. P. J. Murphy, Queen's College, Cork; T. Murphy, Queen's College, Cork; R. J. Murray, University College, Dublin; J. C. Nixon, Queen's College, Galway; M. J. O'Kane, University College, Dublin; J. O'Neill, University College, Dublin; W. A. Osborne, Queen's College, Belfast; H. Paterson, Queen's College, Cork; R. S. Ryce, Queen's College, Cork; B. H. Shaw, Queen's College, Cork; W. S. Smyth, Queen's College, Belfast! W. M. H. Spiller, Queen's College, Belfast; R. C. Stuart, Queen's College, Belfast; A. G. Sutton, Queen's College, Cork; J. V. G. Tighe, University College, Dublin; J. F. Whelan, University College, Dublin.

The following may present themselves for the Honour Examinations as below: S. T. Begg, in Zoology, Physics, and Chemistry; E. O. B. Carbery, in Botany; J. Crean, in Botany and Chemistry; R. V. Donnellan, in Botany; R. P. Farnan, in Zoology, Botany, Chemistry, and Physics; G. K. Finlay, in Botany, Chemistry, and Physics; T. Finucane, in Botany, Zoology, and Physics; S. F. Floyd, in Zoology and Physics; H. J. Fryer, in Zoology and Chemistry; P. J. Hamilton, in Botany and Zoology; J. Harvey, in Chemistry; J. Lennon, in Chemistry; J. F. Little, in Botany; A. A. F. McArdle, in Zoology, Chemistry, and Physics; J. McArdle, in Botany, Chemistry, and Physics; A. L. McCully, in Chemistry and Physics; H. J. McNabb, in Botany; L. T. Moore, in Botany and Zoology; W. W. Moore, B.A., in Zoology and Chemistry; M. R. Morrissey, in Physics; J. P. J. Murphy, in Zoology; T. Murphy, in Physics; R. J. Murray, in Botany, Zoology, Chemistry, and Physics; M. J. O'Kane, in Botany and Zoology; J. O'Neill, in Chemistry; W. A. Osborne, in Botany, Zoology, Chemistry, and Physics; H. Paterson, in Botany and Physics; R. S. Ryce, in Physics and Chemistry; B. H. Shaw, in Botany; W. M. H. Spiller, in Chemistry.

**Examining Board in England by the Royal Colleges of Physicians and Surgeons.**

The following gentlemen passed the Second Examination of the Board in Anatomy and Physiology at a meeting of the Examiners on Monday, July 4th: T. S. Hartley, W. F. Rawson, E. G. Storrs, and P. V. Fry, students of Yorkshire College, Leeds; W. R. Walton, of University College, Liverpool; T. F. Woolley, W. Allport, W. S. Willmore, G. H. E. Bekenn, E. B. Bostock, G. E. Atkins, and H. W. Pepper, of Queen's College, Birmingham; Subhan Ali, of Lahore Medical School, Punjab; E. W. Battle, of Owens College, Manchester; F. H. Humphris, of Edinburgh University and Mr. Cooke's School of Anatomy and Physiology; F. F. Noonan, of Melbourne and Edinburgh Universities; A. Lockhart and Isaac Wood, of Queen's College, Kingston, Canada.

Passed in Anatomy only: C. A. Phillips, E. H. Phillips, and G. T. Myrtle, of Yorkshire College, Leeds; H. W. Lloyd, of University College, Liverpool; E. W. Ormerod, of Bristol Medical School; and H. A. L. Banham, of Sheffield Medical School.

Passed in Physiology only: G. R. Sparrow, of University College, Liverpool.

Twelve candidates were referred in both subjects, one in anatomy, and six in physiology only.

Passed in Anatomy and Physiology on Tuesday, July 5th: P. Macaulay, of Yorkshire College, Leeds; R. Hewlett Hayes, of Dublin and Guy's Hospital; L. N. Pentreath, of St. Thomas's Hospital; D. L. Clay and J. Hepworth, of Owens College, Manchester; A. K. White, of Dublin; W. H. Brown, D. Horwich, W. G. Thomas, and W. H. G. Wilkes, of Queen's College, Birmingham; T. J. Chidley, of Dublin and Mr. Cooke's School of Anatomy and Physiology.

Passed in Anatomy only: W. Cooper, J. L. Elliott, and A. T. Lachmore, of Yorkshire College, Leeds; J. T. Barritt and R. Marshall, of Owens College, Manchester; F. A. W. Quay, of Trinity College, Toronto; B. P. O'Neill, of Guy's Hospital; H. P. Kennard, of St. Thomas's Hospital; J. A. K. Griffiths, of University College; E. A. McAnally, of St. George's Hospital and Mr. Cooke's School of Anatomy and Physiology; and A. C. Greenwood, of Middlesex Hospital.

Passed in Physiology only: W. Archer and W. G. Parkinson, of Yorkshire College, Leeds; T. W. W. Bovey, of Bristol Medical School; F. A. L'E. Burges and J. W. Farndale, of Queen's College, Birmingham; T. H. Agnew, of University College, Liverpool; H. J. Heginbotham, J. Worthington, and S. Crossley, of Owens College, Manchester.

Nine candidates were referred in both subjects, four in Anatomy only, and ten in Physiology only.

Passed in Anatomy and Physiology on Wednesday, July 6th: J. Hora, T. H. B. Yorath, and H. D. Packer, of Guy's Hospital; J. C. Padwick, G. Miller, and S. C. Hounsfield, of St. Bartholomew's Hospital; L. W. Burrow, of St. Thomas's Hospital; and F. A. J. R. Brooke, of London Hospital.

Passed in Anatomy only: J. L. F. de Gannes and C. W. Smith, of University College; P. R. Wallis, of University College and Mr. Cooke's School of Anatomy and Physiology; C. A. A. Coulthard, of St. George's Hospital; C. F. Stileman, of St. George's Hospital and Mr. Cooke's School of Anatomy and Physiology; T. H. Parker and H. P. Cox, of King's College; E. H. Tipper, of Guy's Hospital; P. L. Blaber, of St. Thomas's Hospital; and A. P. Birch, of Middlesex Hospital and Mr. Cooke's School of Anatomy and Physiology.

Passed in Physiology only: E. E. Crowther, of Yorkshire College, Leeds; G. V. Miller and F. P. Duncan, of University College; W. S. Hopkins, of St. Bartholomew's Hospital; H. A. Burridge, of King's College; and C. C. Jenkins, of Guy's Hospital.

